# Bioactivity-guided metabolomics reveals discriminant cytotoxic signatures in *Siparuna guianensis*

**DOI:** 10.1007/s11306-026-02461-1

**Published:** 2026-05-29

**Authors:** Salva Asghar, Giovanni B. Bevilaqua, Gracimerio J. Guarneire, Samira C. C. Ludgero, Sandra B. R. Castro, Alessandra P. Carli, Caio César S. Alves, Boniek Gontijo, Danielle F. Dias, Marisi G. Soares, Nerilson M. Lima

**Affiliations:** 1 Institute of Chemistry, Federal University of Alfenas, Alfenas, MG 37130-001 Brazil; 2 Chemistry Institute, Federal University of Goiás, Goiânia, Goiás 74690-900 Brazil; 3 Faculty of Medicine, Universidade Federal Dos Vales Do Jequitinhonha E Mucuri, Teófilo Otoni, MG 39803-371 Brazil

**Keywords:** Cancer, Bioactivity-guided fractionation, Untargeted metabolomics, High-resolution mass spectrometry (HRMS), Aporphine alkaloids

## Abstract

**Introduction:**

Natural products remain a privileged source of structurally diverse bioactive compounds with potential for the development of safer and more selective anticancer agents.

**Objectives:**

In this study, a bioactivity-guided untargeted metabolomics approach was applied to investigate the cytotoxic chemical space of *Siparuna guianensis*.

**Methods:**

The hydroethanolic leaf extract and solvent-partitioned fractions (hexane, ethyl acetate, butanol, and aqueous) were evaluated for cytotoxic activity against MCF-7, 4T1, and MDA-MB-231 breast cancer cell lines, followed by metabolomic profiling using HPLC–HRMS.

**Results:**

Cytotoxicity was predominantly associated with low- and intermediate-polarity fractions, which were classified as active and subsequently compared with inactive samples using chemometric methods. Structural annotation supported by spectral libraries enabled MSI level 2–3 annotation of 60 metabolites. Alkaloids and flavonoids were proportionally enriched in cytotoxic fractions despite the overall dominance of terpenoids. Multivariate and univariate statistical analyses demonstrated clear metabolic discrimination between active and inactive groups. Integration of VIP scores, Volcano analysis, and ROC-based prioritization identified isoquinoline and aporphine alkaloids, together with glycosylated flavonoids, as the principal contributors to cytotoxicity. The alkaloid norglaucine emerged as a key discriminant feature in the VIP–Volcano intersection, consistent with its previously reported cytotoxic activity against multiple tumor cell lines. Consensus discriminant ions included *m/z* 330.170 and 296.12, both showing high discriminative potential.

**Conclusions:**

This study suggests a strong association between metabolomic composition and cytotoxic activity in *S. guianensis*, highlighting isoquinoline-derived scaffolds as promising candidates for future isolation and mechanistic investigation while demonstrating the power of metabolomics-guided strategies for natural product discovery.

## Introduction

Cancer remains a major global health burden, demanding the continuous development of safer and more effective therapeutic strategies characterized by improved selectivity and reduced systemic toxicity. In this scenario, natural products remain a privileged source of structurally diverse bioactive molecules, frequently providing unique chemical scaffolds with enhanced therapeutic potential and fewer adverse effects compared to purely synthetic compounds. Within this context, *Siparuna brasiliensis* (Spreng.) ADC. (“negramina”), a species native to the Brazilian Atlantic Forest and Cerrado and traditionally used by indigenous communities, has emerged as a promising pharmacological resource. Evidence from related species reinforces this potential, as studies with *Siparuna guianensis* essential oils demonstrated cytotoxic activity against tumor models, including MCF-7 breast cancer cells, highlighting that *Siparuna*-derived metabolomes may harbor anticancer-relevant chemistry, although species- and fraction-specific investigations remain essential to uncover the active chemical space (Avanci-Júnior et al., 2025). Phytochemical investigations further support this anticancer relevance, as liriodenine, an oxoaporphine alkaloid isolated from *S. guianensis*, induces apoptosis and suppresses cellular proliferation in MCF-7 cells (Li et al., 2017), while ( −)-anonaine promotes tumor cell death through Bax/caspase-mediated and ROS-dependent mechanisms (Chen et al., 2008). Similarly, the isoquinoline alkaloid norglaucine, identified in methanolic fruit extracts, exhibits cytotoxic activity against multiple tumor cell lines, including HepG2, K562, HL-60, and B16-F10 (Menezes et al., 2016).

The comprehensive characterization of bioactive plant metabolomes increasingly depends on advanced analytical strategies capable of navigating chemical complexity beyond traditional isolation workflows. Although compound purification remains essential for definitive structural confirmation, it is often labor-intensive and inefficient when applied to chemically diverse botanical matrices. In this scenario, untargeted metabolomics has emerged as a transformative approach, combining high-resolution analytical platforms with bioinformatics-driven data interpretation to accelerate bioactive compound discovery. Among available technologies, liquid chromatography coupled with high-resolution mass spectrometry (LC–HRMS) stands out due to its sensitivity, selectivity, and capacity to simultaneously detect chemically diverse metabolites at trace levels within complex extracts (Domínguez et al., 2020; Wolfender et al., 2018). Nevertheless, the extensive datasets generated by HRMS analyses require robust computational and statistical frameworks to enable reliable metabolite annotation and biologically meaningful interpretation (Chang et al., 2021).

To overcome the analytical and interpretative challenges inherent to large-scale untargeted metabolomics datasets, molecular networking (MN) has emerged as a robust computational framework for organizing and visualizing MS/MS data based on spectral similarity. Implemented through platforms such as the Global Natural Products Social Molecular Networking (GNPS), MN enables rapid dereplication, molecular family clustering, and annotation propagation across structurally related metabolites (Pilon et al., 2021; Quinn et al., 2017). Feature-based molecular networking (FBMN) further refines this strategy by integrating chromatographic alignment, isotope patterns, and relative quantification, thereby improving discrimination among isomers and closely related compounds (Nothias et al., 2020). Complementary in silico tools expand annotation depth through substructure recognition, automated class prediction, biosynthetic inference, and machine-learning–based molecular fingerprinting (Dührkop et al., 2015; Ernst et al., 2019; Santos et al., 2023; van Der Hooft et al., 2016). Nevertheless, metabolite identification in mass spectrometry–based studies frequently remains restricted to MSI confidence levels 2 and 3 (Sumner et al., 2007), particularly when features derive predominantly from MS^1^ acquisition, which provides broader metabolome coverage but limited structural resolution. To mitigate this limitation, compositional descriptors derived from molecular formulas such as Van Krevelen diagrams have been increasingly incorporated as complementary chemoinformatic strategies for class-level annotation and structural interpretation (Brockman et al., 2018; Krevelen, 1950; Lima et al., 2025), enabling improved discrimination of metabolite classes and biosynthetic scaffolds within complex plant metabolomes.

Despite the expanding arsenal of metabolites annotated through advanced analytical and computational platforms, biological interpretation remains a critical step in untargeted metabolomics. In this context, chemometrics plays a central role by transforming high-dimensional spectral datasets into statistically robust and biologically meaningful information. Beyond mere metabolite detection, untargeted metabolomics aims to reduce data dimensionality without loss of relevant variance, uncover global compositional trends, discriminate biological groups, and prioritize activity-associated features—objectives that fundamentally depend on multivariate statistical modeling (García-Pérez et al., 2024). Among the most widely applied approaches, Principal Component Analysis (PCA) enables unsupervised exploration of intrinsic data structure by capturing dominant variance patterns while minimizing noise and redundancy (Bro & Smilde, 2014). Complementarily, Partial Least Squares–Discriminant Analysis (PLS-DA) incorporates class information to maximize covariance between metabolite features and predefined biological groups, enhancing discriminative power in complex datasets (Lee et al., 2018). Together, these complementary tools provide a robust framework for identifying metabolite patterns associated with biological phenotypes and prioritizing chemically and biologically relevant candidates within complex plant metabolomes.

In this study, a bioactivity-guided untargeted metabolomics approach was applied to investigate the cytotoxic chemical space of *Siparuna guianensis* leaf extracts and solvent-derived fractions against breast cancer cell lines. LC–Orbitrap-HRMS analysis combined with GNPS molecular networking and in silico annotation tools enabled metabolite dereplication and MSI level 2–3 structural annotation. Multivariate and univariate chemometric analyses were used to discriminate active and inactive samples, while biomarker prioritization integrating OPLS-DA-derived VIP and regression scores allowed the selection of isoquinoline alkaloids and polyphenolic metabolites associated with cytotoxic activity.

## Materials and methods

### Plant material extraction and LC–HRMS/MS analysis

Plant material was collected in September 2024 in the municipality of Alfenas, Minas Gerais, Brazil, within the Atlantic Forest biome. Leaves and branches of *Siparuna guianensis* were air-dried at room temperature and subsequently milled. The dried plant material (100 g) was extracted at room temperature using a hydroethanolic solvent system (ethanol/water, 70:30, *v/v*), applying a sample-to-solvent ratio of 1:1 (*w/v*). Extractions were performed in three consecutive cycles (100 mL per cycle) under constant agitation to maximize metabolite recovery. The extracts were concentrated under reduced pressure using a rotary evaporator and freeze-dried to yield the crude extracts. The crude extract was subjected to liquid–liquid extraction using solvents of increasing polarity (hexane, ethyl acetate, *n*-butanol, and water), yielding the respective fractions after solvent removal under reduced pressure. Prior to analysis, the extracts were reconstituted in methanol at a final concentration of 300 µg mL⁻^1^ and filtered before injection.

Chromatographic separations were carried out on an HPLC 1220 Infinity II system (*Agilent Technologies*) coupled to a Q-Exactive hybrid quadrupole-Orbitrap high-resolution mass spectrometer (*Thermo Scientific*) equipped with an electrospray ionization (ESI) source. An InfinityLab Poroshell 120 EC-C18 column (4.6 × 100 mm, 2.7 µm; Agilent) was employed for all analyses. The mobile phase consisted of water containing 0.1% formic acid (A) and methanol (B), delivered under a linear gradient starting at 5% B, increasing to 100% B over 40 min, followed by an isocratic hold at 100% B for 10 min. The system was then returned to initial conditions over 10 min for column re-equilibration. The flow rate was maintained at 0.3 mL min⁻^1^, the injection volume was 25 µL, and the column temperature was set to 35 °C.

Mass spectrometric data were acquired in positive ionization mode using full MS combined with data-dependent MS/MS (dd-MS^2^). The ESI source parameters were set as follows: spray voltage, 3.5 kV; capillary temperature, 250 °C; S-lens RF level, 60 V; sheath gas flow rate, 47 L min⁻^1^; auxiliary gas flow rate, 11 L min⁻^1^. Full-scan mass spectra were recorded over a *m/z* range of 100–1200. The five most intense precursor ions per scan were automatically selected for fragmentation (Top5, loop count 5), using stepped normalized collision energies of 20, 30, and 35 eV. The resolving power was set to 140,000 (at *m/z* 200) for full MS scans and 70,000 for MS/MS acquisitions.

### Putative metabolite annotation

Raw LC–HRMS/MS files were converted to the mzML format using MSConvert (ProteoWizard) before data processing in MZmine 2 (version 2.53). Mass detection for MS^1^ and MS^2^ spectra was performed in centroid mode, applying noise thresholds of 1.0 × 10⁶ and 1.0 × 10^4^, respectively. Chromatograms were constructed using the ADAP chromatogram builder, which requires a minimum of five consecutive scans, a group intensity threshold of 3.0 × 10⁶, and a *m/z* tolerance of 10 ppm. Peak deconvolution was conducted using the baseline cut-off algorithm, with a minimum peak height of 3.0 × 10⁶, peak duration between 0.02 and 2.0 min, and a baseline level of 1.0 × 10⁶. Isotopic features were grouped using a *m/z* tolerance of 10 ppm, retention time tolerance of 0.05 min, and a maximum charge state of two. Feature alignment across samples was achieved with the Join Aligner algorithm, applying identical *m/z* and retention time tolerances. Gap filling was subsequently performed using the peak finder approach, and only features associated with MS/MS spectra were retained for downstream analysis.

Molecular annotation was performed within the Global Natural Products Social Molecular Networking (GNPS) ecosystem, using both Classical Molecular Networking—MN (Wang et al., 2016) and Feature-Based Molecular Networking—FBMN (Nothias et al., 2020) workflows. Experimental MS/MS spectra were matched against curated GNPS spectral libraries, which classify reference spectra according to annotation confidence levels. Additional dereplication and annotation support was obtained through integrated in silico tools, including DEREPLICATOR + (Mohimani et al., 2018), Network Annotation Propagation—NAP (da Silva et al., 2018), MolDiscovery (Cao et al., 2021), MS2LDA (van Der Hooft et al., 2016), MolNetEnhancer (Ernst et al., 2019), and SIRIUS (Dührkop et al., 2015). Retention time behavior and UV absorption profiles were also considered as complementary orthogonal information to support structural hypotheses.

Accurate molecular formulas were generated using Thermo Scientific Xcalibur software based on high-resolution mass measurements and isotopic pattern fitting. Only molecular formulas presenting mass errors below 2 ppm were retained. To support class-level annotation (MSI level 3), elemental ratios and unsaturation indices were systematically explored. Van Krevelen diagrams were constructed using hydrogen-to-carbon (H/C) and oxygen-to-carbon (O/C) atomic ratios highlighting the CHNO regions, allowing visualization of compositional trends and chemical class distributions.

### Multivariate and univariate statistical analyses

To initially assess the distribution and overlap of metabolites across samples, feature intersections were evaluated using UpSet plot analysis. This approach enables robust visualization of shared and unique metabolite features across multiple groups. Multivariate and univariate statistical analyses were employed to comprehensively investigate metabolic variations between the experimental groups. To ensure that the observed metabolic discrimination was associated with cytotoxic activity rather than solvent polarity alone, samples were classified into active and inactive groups based on biological response (cell viability < 70%), independent of extraction solvent. This strategy minimizes bias introduced by polarity-driven chemical segregation and enables biologically driven metabolomic interpretation. Initially, principal component analysis (PCA) was applied as an unsupervised method to evaluate intrinsic data structure, detect outliers, and assess overall sample clustering. Supervised models, including partial least squares discriminant analysis (PLS-DA) and orthogonal PLS-DA (OPLS-DA), were subsequently used to maximize group discrimination and to isolate predictive variation related to cytotoxic effects from orthogonal, non-relevant variability. Regression (REG) coefficients were used to quantify the direction and magnitude of each metabolite’s contribution to class separation, enabling biologically oriented prioritization of discriminant compounds. Hierarchical clustering analysis (HCA) and heatmap visualization were performed using sample averages and the 50 most discriminant features selected by t-test/ANOVA, employing Euclidean distance and Ward’s linkage, to highlight patterns of metabolite abundance across samples. Differential features were further identified using Volcano Plot analysis based on a fold change ≥ 4 and p-value ≤ 0.01, followed by biomarker evaluation through receiver operating characteristic (ROC) curve analysis to assess the discriminatory performance of individual and combined metabolites. To enhance biological interpretation, enrichment analysis and metabolic pathway analysis integrating pathway over-representation and topology information were conducted using the Mummichog algorithm in MetaboAnalyst 5.0, enabling direct mapping of significant *m/z* features to KEGG pathways (*Arabidopsis thaliana*), with a mass tolerance of 5 ppm, primary ions enforced, and a significance threshold of p ≤ 0.01. For univariate statistical analysis, the data matrix generated from MZmine processing was subjected to chemometric evaluation using the MetaboAnalyst platform (version 6.0). The dataset was initially normalized, followed by autoscaling and square root transformation.

### Cell viability of 4T1, MCF-7 and MDA-MB-231 treated with crude extract and fractions from *S. guianensis*

To cell culture, the samples stock solutions were made from sterile RPMI and stored at -80 °C until use. 4T1, MCF-7 and MDA-MB-231 cancer cell lines were maintained in culture bottles containing supplemented RPMI-1640 medium (1% non-essential amino acids, 100 μg mL^−1^ streptomycin and penicillin, and 5% fetal bovine serum) in a humidified atmosphere of 5% CO_2_ at 37 °C. After reaching confluence, the bottles were trypsinized, and the cells were plated in 96-well plates at the concentration of 2 × 10^4^ mL^−1^ cells. Cells were incubated in a humidified atmosphere of 5% CO_2_ at 37 °C in the presence of the SGL2.65 ext. bruto, EtoAC fr. SGL, Aquous ffr. SGL, Butanol fr. SGL, Hexane fr. SGL, Alkaloid, or Branchs at 1:100, 1:1,000, 1:10,000, or 1:100,000 for 48 h.

Cell viability was measured using the MTT [3-(4,5-dimethylthiazolyl-2)-2,5-diphenyltetrazolium bromide] assay in culture of not stimulated cells. After 48 h of culture, the supernatant was removed, and the cells were incubated with 100 μL of supplemented RPMI and 10 μL of MTT (5 mg mL^−1^) for 4 h in a humidified atmosphere of 5% CO_2_ at 37 °C. After this period, the supernatant was withdrawn from the wells without any change in the precipitate. The formed formazan crystals were then dissolved by adding 100 μL of DMSO in each well. The complete solubilization was done by a slight agitation of the plates. The optical density was measured at 570 nm (Biochrom), and the viability (%) was obtained by the formula [(X1/X2) × 100), considering X1 and X2 the mean optical density in wells of treated cells and cells untreated, respectively. Treatments that reduce the viability below 70% were considered cytotoxic.

Concerning the statistical analysis, the results are presented as the mean ± standard error and are representative of three independent experiments.

## Results and discussion

### Cytotoxic profiling of *S. guianensis* fractions against breast cancer cell lines

**Fig. 1:**
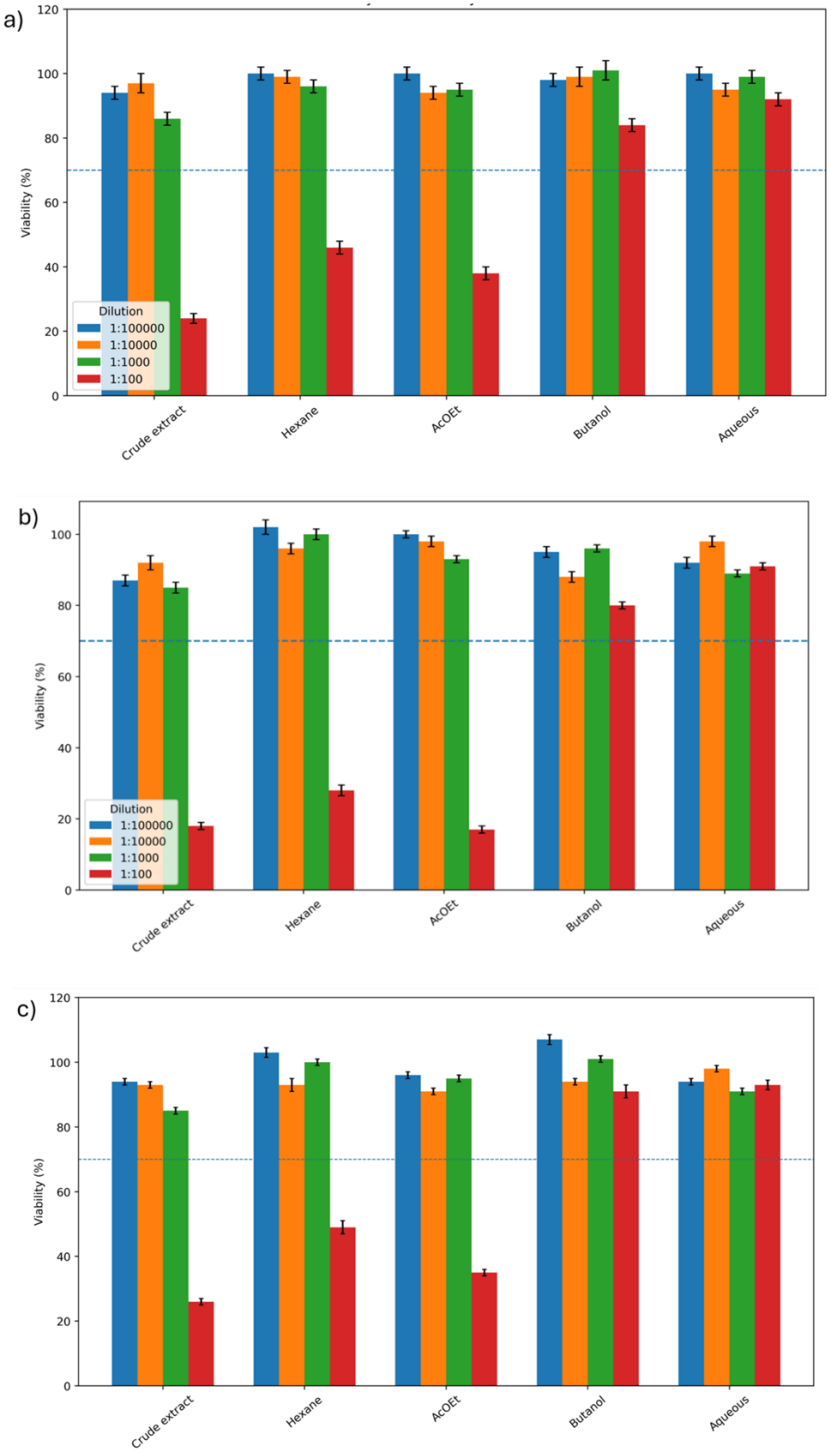
Cytotoxic activity of *Siparuna guianensis* crude extract and solvent fractions against breast cancer cell lines. **a** MCF-7, **b** 4T1, and **c** MDA-MB-231 cells treated with the crude extract and hexane, ethyl acetate (AcOEt), butanol, and aqueous fractions at serial dilutions (1:100,000, 1:10,000, 1:1000, and 1:100). Cell viability (%) was determined after treatment and is presented as mean ± standard deviation. The blue horizontal line indicates the activity threshold (70% viability) used to stratify samples into active and non-active groups for subsequent metabolomic and chemometric analyses

The cytotoxic activity of the crude extract and solvent-derived fractions of *Siparuna guianensis* was evaluated against MCF-7, 4T1, and MDA-MB-231 breast cancer cell lines, serving as the biological criterion for subsequent metabolomic and chemometric stratification into active and non-active groups (Fig. 1a–c). Cytotoxicity was strongly concentration- and polarity-dependent, with the most pronounced reductions in viability observed at the highest tested concentration (1:100 dilution).

In MCF-7 cells **(**Fig. 1a**)**, the crude extract exhibited the strongest cytotoxic effect, followed by the hexane and ethyl acetate fractions, whereas butanol and aqueous fractions showed limited activity. A similar trend was observed for 4T1 cells **(**Fig. 1b**)**, where low-to-medium polarity fractions demonstrated greater bioactivity compared to polar fractions. In MDA-MB-231 cells **(**Fig. 1c**)**, overall sensitivity was lower; however, concentration-dependent effects remained evident, particularly for the crude extract and less polar fractions.

Collectively, these results indicate that bioactive metabolites are enriched mainly in the crude extract and low-to-intermediate polarity fractions, providing the biological foundation for downstream metabolomic discrimination and biomarker discovery analyses.

### Metabolomic profiling and chemical annotation reveal cytotoxicity-associated metabolic signatures

**Fig. 2:**
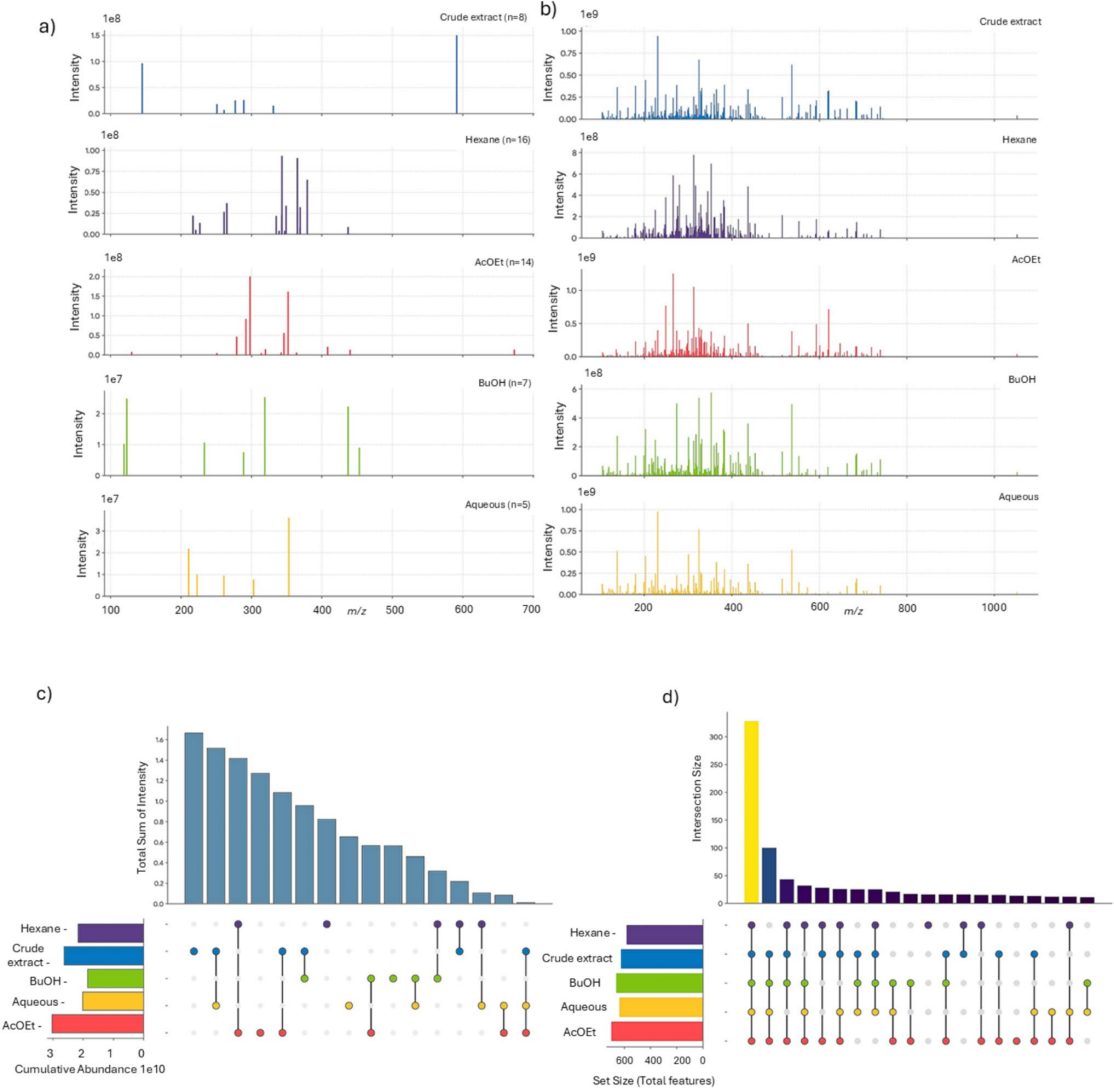
Feature distribution across liquid–liquid fractions of *Siparuna guianensis*. **a** Overlaid LC-HRMS mass spectra showing the global chemical composition of the crude extract and solvent-derived fractions, **b** Stacked spectra displaying ions exclusively detected in each fraction, **c** Feature-based UpSet plot illustrating the number of shared and unique molecular features among fractions, **d** Abundance-weighted UpSet analysis showing cumulative signal intensity distribution across fractions, providing insight into the contribution of dominant metabolites to inter-fraction chemical differentiation

The metabolomic composition of the crude extract and solvent-partitioned fractions was initially investigated through direct visual inspection and spectral comparison of LC-HRMS profiles, focusing on qualitative (presence/absence of ions) and semi-quantitative (relative abundance) differences among samples (Fig. 2a). The overlaid raw mass spectra revealed pronounced variations in ion distribution across the *m/z* range, indicating that liquid–liquid partitioning effectively redistributed metabolites according to solvent polarity. While several ions were shared among fractions, clear differences in signal density and intensity patterns suggested progressive enrichment of chemically distinct metabolite subsets, particularly between apolar (hexane) and polar (Butanol and aqueous) phases.

To refine this interpretation, only exclusive ions detected uniquely in each fraction were subsequently visualized (Fig. 2b). This approach highlighted a marked chemical specialization resulting from the extraction workflow, demonstrating that each solvent selectively concentrated specific molecular features. The hexane and AcOEt (ethyl acetate) fractions exhibited a higher number of exclusive signals within intermediate *m/z* regions, consistent with enrichment of moderately lipophilic metabolites, whereas BuOH (butanol) and aqueous fractions displayed fewer but chemically distinct ions, suggesting preferential recovery of polar constituents. The crude extract retained unique features absent from all derived fractions, indicating partial metabolite loss or redistribution during partitioning.

Complementarily, a feature-based UpSet analysis was performed to quantitatively evaluate inter-fraction overlap (Fig. 2c). This analysis demonstrated that although a subset of metabolites remained shared across multiple fractions, a substantial proportion of features were fraction-specific, confirming the efficiency of liquid–liquid extraction in reducing chemical redundancy and improving metabolome resolution. The large number of unique intersections observed for individual fractions supports polarity-guided chemical segregation rather than simple dilution or random redistribution of metabolites.

Because biological activity is frequently driven by dominant constituents rather than feature counts alone, an additional abundance-weighted UpSet analysis was conducted (Fig. 2d). When cumulative signal intensity was considered, the distribution pattern shifted markedly, revealing that high-abundance metabolites were not uniformly shared among fractions. Instead, specific solvent systems concentrated chemically dominant ions, reinforcing the effectiveness of the extraction strategy in generating metabolically differentiated samples. Together, these complementary analyses demonstrate that liquid–liquid partitioning produced chemically orthogonal fractions, establishing a robust foundation for subsequent chemometric discrimination and metabolomics-guided identification of cytotoxic biomarkers.

**Fig. 3:**
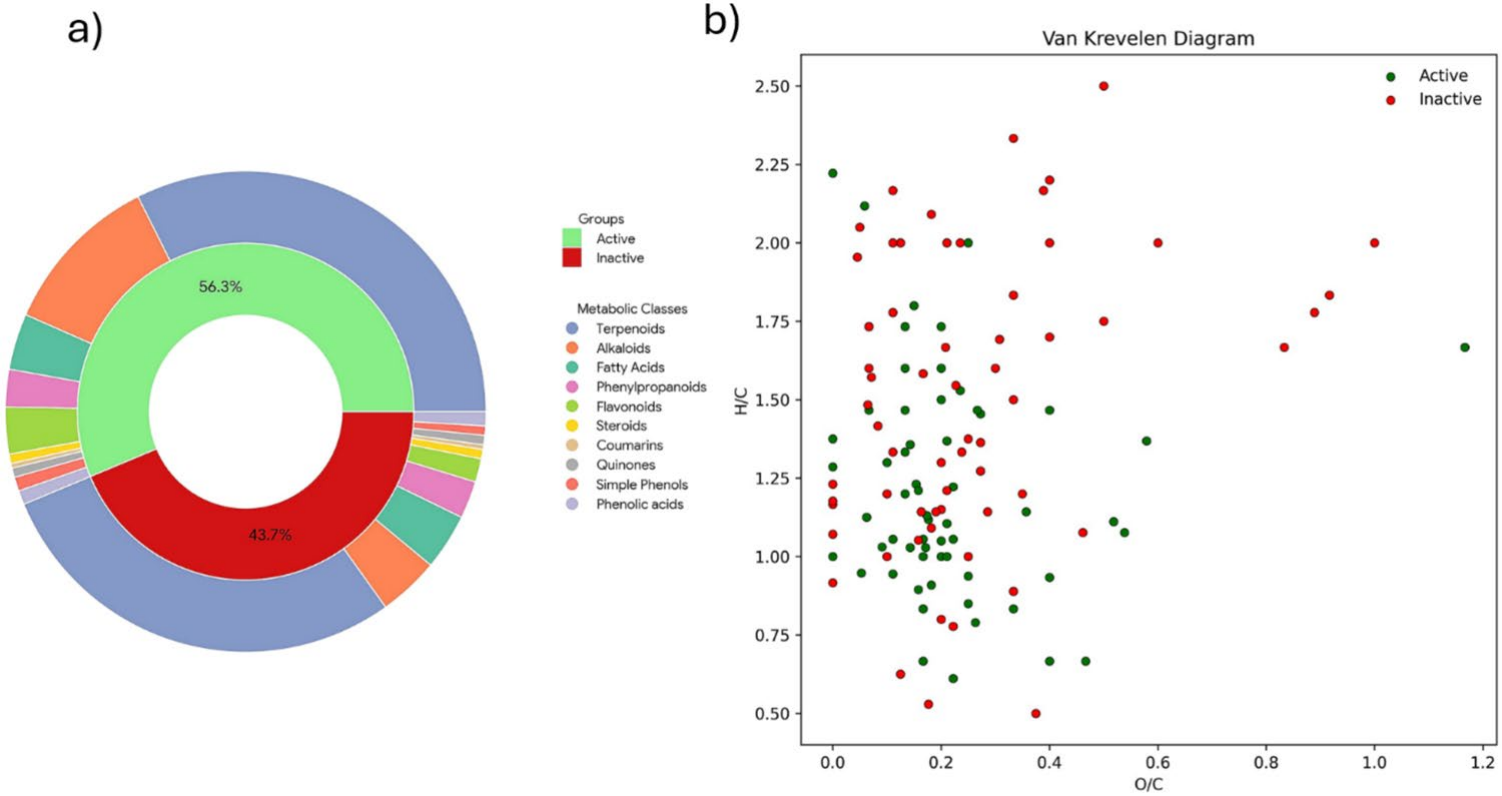
MSI level 3 structural annotation and chemical space distribution of metabolites associated with cytotoxic activity. **a** Donut plot showing the relative abundance of annotated metabolic classes (terpenoids, alkaloids, fatty acids, flavonoids, phenylpropanoids, phenolic acids, simple phenols, steroids, quinones, and coumarins) in active and inactive groups. **b** Van Krevelen diagram (H/C vs. O/C ratios) illustrating the molecular compositional domains occupied by metabolites according to bioactivity classification

Structural annotation performed at MSI confidence levels 2 and 3, supported by spectral library matching and in silico classification workflows, enabled a comprehensive characterization of the secondary metabolite landscape across the active and inactive groups. The chemical class distribution (Fig. 3a) revealed a metabolome strongly dominated by terpenoids, representing 47.59% of the annotated metabolites in the active group and 55.56% in the inactive group, confirming their pervasive occurrence in *Siparuna* metabolomes. Despite this overall dominance, relevant compositional shifts were observed between biological groups. Alkaloids were markedly enriched in the active samples (24.14%) compared to inactive fractions (12.04%), highlighting this class as a major contributor to cytotoxic activity and consistent with the known antitumoral relevance of isoquinoline alkaloids (aporphine-type alkaloids, benzyl-tetrahydroisoquinolines, and simple tetrahydroisoquinoline) previously reported for the genus (Conegundes et al., 2021 Gomes et al., 2025; Vilanculo et al., 2025). Likewise, flavonoids were proportionally more represented in active fractions (6.90%) than in inactive ones (4.63%), reinforcing their potential role as bioactivity modulators through antioxidant and apoptosis-related mechanisms.

Conversely, inactive samples showed relatively higher proportions of fatty acids (11.11% vs. 8.28%) and phenylpropanoids (7.41% vs. 5.52%), suggesting enrichment in metabolites more associated with structural or primary metabolic functions rather than selective cytotoxic responses. Minor yet consistent contributions from phenolic acids (2.07–2.78%), simple phenols (1.85–2.07%), steroids (1.38–1.85%), quinones (1.38–1.85%), and coumarins (0.69–0.93%) further illustrate the chemical diversity distributed between groups.

Complementarily, the Van Krevelen diagram (Fig. 3b) expanded metabolite classification beyond structural annotation by exploring elemental composition patterns through H/C and O/C ratios, enabling visualization of chemical space occupation associated with bioactivity. Active samples preferentially clustered within regions characteristic of moderately oxygenated and hydrogen-rich metabolites, compatible with alkaloids, flavonoids, and oxygenated terpenoids, whereas inactive samples exhibited broader dispersion toward higher oxidation domains. This compositional segregation reinforces the hypothesis that cytotoxic activity is not driven solely by metabolite abundance but by specific chemical families occupying defined regions of molecular compositional space, highlighting the complementarity between annotation-based classification and elemental ratio-driven chemoinformatic analysis (Lima et al., 2025).

An untargeted metabolomics workflow was employed to comprehensively characterize the chemical composition of *S. guianensis*, combining dereplication strategies based on MS/MS spectral similarity with advanced computational annotation tools. Metabolite annotation at MSI confidence level 2 was achieved through the integration of experimental spectral libraries available within the GNPS ecosystem and in silico fragmentation prediction using the SIRIUS platform. Candidate structures were supported by accurate mass–based molecular formula assignment, diagnostic fragmentation pattern analysis, chromatographic behavior (retention time and UV absorption profiles), and critical manual inspection of structural analogues proposed by spectral matching algorithms. Additionally, annotations were prioritized considering chemophenetic consistency within the Siparunaceae family, strengthening biological plausibility. In total, 60 metabolites were putatively annotated, comprising one quinone, two coumarins, three phenylpropanoids, eleven flavonoids, and forty-three alkaloids, highlighting the strong enrichment of nitrogen-containing specialized metabolites in the metabolome.

Table 1 compiles all putatively annotated metabolites, including compound names, *m/z* values, molecular formulas, retention times, chemical structures, and metabolite class assignments.

**Table 1:** Annotated compounds in *Siparuna guianensis* samples by LC–HRMS/MS, including *m/z* value, compound name, molecular formula, proposed chemical structure and metabolite class

*m/z*	Compound name	Molecular formula	Chemical structure	Metabolite class
282.15	2-methoxy-6-methyl-5,6,6a,7-tetrahydro-4H-dibenzo[de,g]quinolin-1-ol	C_18_H_19_NO_2_		Alkaloid
280.13	7-methyl-6,7,7a,8-tetrahydro-5H-[1,3]dioxolo[4',5':4,5]benzo[1,2,3-de]benzo[g]quinoline	C_18_H_17_NO_2_		Alkaloid
308.12	(7R)-6,7,7a,8-Tetrahydro-7-acetyl-5H-benzo[g]-1,3-benzodioxolo[6,5,4-de]quinoline	C_19_H_17_NO_3_		Alkaloid
340.15	10,11-dimethoxy-7-methyl-6,7,7a,8-tetrahydro-5H-[1,3]dioxolo[4',5':4,5]benzo[1,2,3-de]benzo[g]quinoline	C_20_H_21_NO_4_		Alkaloid
356.18	1,2,9,10-tetramethoxy-6-methyl-5,6,6a,7-tetrahydro-4H-dibenzo[de,g]quinoline	C_21_H_25_NO_4_		Alkaloid
372.18	1,2,9,10-tetramethoxy-6-methyl-5,6,6a,7-tetrahydro-4H-dibenzo[de,g]quinolin-8-ol	C_21_H_25_NO_5_		Alkaloid
358.16	(S)-1,2,9,10-tetramethoxy-5,6,6a,7-tetrahydro-4H-dibenzo[de,g]quinolin-8-ol	C_20_H_23_NO_5_		Alkaloid
328.15	(S)-1,2,10-trimethoxy-5,6,6a,7-tetrahydro-4H-dibenzo[de,g]quinolin-9-ol	C_19_H_21_NO_4_		Alkaloid
282.14	1,2-dimethoxy-5,6,6a,7-tetrahydro-4H-dibenzo[de,g]quinoline	C_18_H_19_NO_2_		Alkaloid
312.16	1,2,10-trimethoxy-5,6,6a,7-tetrahydro-4H-dibenzo[de,g]quinoline	C_19_H_21_NO_3_		Alkaloid
326.17	1,2,10-trimethoxy-6-methyl-5,6,6a,7-tetrahydro-4H-dibenzo[de,g]quinoline	C_20_H_23_NO_3_		Alkaloid
268.13	1-methoxy-5,6,6a,7-tetrahydro-4H-dibenzo[de,g]quinolin-2-ol	C_17_H_17_NO_2_		Alkaloid
310.14	3-methoxy-6-methyl-5,6,6a,7-tetrahydro-4H-[1,3]dioxolo[4',5':4,5]benzo[1,2-g]benzo[de]quinoline	C_19_H_19_NO_3_		Alkaloid
312.16	1,10-dimethoxy-6-methyl-5,6,6a,7-tetrahydro-4H-dibenzo[de,g]quinolin-2-ol	C_19_H_21_NO_3_		Alkaloid
312.12	11-methoxy-6,7,7a,8-tetrahydro-5H-[1,3]dioxolo[4',5':4,5]benzo[1,2,3-de]benzo[g]quinolin-10-ol	C_18_H_17_NO_4_		Alkaloid
280.13	7-methyl-6,7,7a,8-tetrahydro-5H-[1,3]dioxolo[4',5':4,5]benzo[1,2,3-de]benzo[g]quinoline	C_18_H_17_NO_2_		Alkaloid
266.11	6,7,7a,8-tetrahydro-5H-[1,3]dioxolo[4',5':4,5]benzo[1,2,3-de]benzo[g]quinoline	C_17_H_15_NO_2_		Alkaloid
296.12	11-methoxy-6,7,7a,8-tetrahydro-5H-[1,3]dioxolo[4',5':4,5]benzo[1,2,3-de]benzo[g]quinoline	C_18_H_17_NO_3_		Alkaloid
314.17	6-methoxy-1-(4-methoxybenzyl)-2-methyl-1,2,3,4-tetrahydroisoquinolin-7-ol	C_19_H_23_NO_3_		Alkaloid
286.14	1-(4-hydroxybenzyl)-6-methoxy-1,2,3,4-tetrahydroisoquinolin-7-ol	C_17_H_19_NO_3_		Alkaloid
300.16	1-(4-hydroxybenzyl)-6-methoxy-2-methyl-1,2,3,4-tetrahydroisoquinolin-7-ol	C_18_H_21_NO_3_		Alkaloid
180.10	(S)-1-methyl-1,2,3,4-tetrahydroisoquinoline-6,7-diol	C_10_H_13_NO_2_		Alkaloid
196.09	(1S)-1-methyl-1,2,3,4-tetrahydroisoquinoline-3,6,7-triol	C_10_H_13_NO_3_		Alkaloid
194.11	(S)-7-methoxy-1-methyl-1,2,3,4-tetrahydroisoquinolin-6-ol	C_11_H_15_NO_2_		Alkaloid
237.10	7,8-dihydroxy-1,1-dimethyl-1,2,3,4-tetrahydroisoquinoline-3-carboxylic acid	C_12_H_15_NO_4_		Alkaloid
342.17	Norglaucine	C_20_H_23_NO_4_		Alkaloid
342.15	(2S)-2-((S)-6,7-dihydroxy-1-methyl-3,4-dihydroisoquinolin-2(1H)-yl)-2-(hydroxymethyl) tetrahydro-2H-pyran-3,4,5-triol	C_16_H_23_NO_7_		Alkaloid
314.17	7,8-dimethoxy-1-(4-methoxybenzyl)-1,2,3,4-tetrahydroisoquinoline	C_19_H_23_NO_3_		Alkaloid
314.17	(S)-6-methoxy-3-(4-methoxybenzyl)-2-methyl-1,2,3,4-tetrahydroisoquinolin-5-ol	C_19_H_23_NO_3_		Alkaloid
300.15	(R)-3-((6,7-dimethoxy-1,2,3,4-tetrahydroisoquinolin-1-yl) methyl) phenol	C_18_H_21_NO_3_		Alkaloid
328.15	4-methoxy-6-(4-methoxybenzyl)-6,7,8,9-tetrahydro- [1,3] dioxolo [4,5-f] isoquinoline	C_19_H_21_NO_4_		Alkaloid
336.08	10,11-dimethoxy-8H-[1,3]dioxolo[4',5':4,5]benzo[1,2,3-de]benzo[g]quinolin-8-one	C_19_H_13_NO_5_		Alkaloid
338.10	10-hydroxy-9-methoxy-5H-[1,3]dioxolo[4,5-g]isoquinolino[3,2-a]isoquinolin-8(6H)-one	C_19_H_15_NO_5_		Alkaloid
352.11	8,9-dimethoxy-11H-[1,3]dioxolo[4,5-h]isoquinolino[2,1-b]isoquinolin-14(12H)-one	C_20_H_17_NO_5_		Alkaloid
358.16	(6aS)-9-hydroxy-1,2,10-trimethoxy-6-methyl-5,6,6a,7-tetrahydro-4H-dibenzo[de,g]quinoline 6-oxide	C_20_H_23_NO_5_		Alkaloid
326.13	(7aS,8S)-10-methoxy-7-methyl-6,7,7a,8-tetrahydro-5H-[1,3]dioxolo[4',5':4,5]benzo[1,2,3-de]benzo[g]quinolin-8-ol	C_19_H_19_NO_4_		Alkaloid
338.10	(4bR,4b1S)-10-methoxy-8,9-dihydro-4bH-[1,3]dioxolo[4',5':4,5]benzo[1,2,3-de]benzo[g]oxazolo[5,4,3-ij]quinolin-6(4b1H)-one	C_19_H_15_NO_5_		Alkaloid
330.17	3-(3-hydroxy-4-methoxybenzyl)-7-methoxy-2-methyl-1,2,3,4-tetrahydroisoquinolin-6-ol	C_19_H_23_NO_4_		Alkaloid
344.18	(R)-5-((6,7-dimethoxy-2-methyl-1,2,3,4-tetrahydroisoquinolin-1-yl) methyl)-2-methoxyphenol	C_20_H_25_NO_4_		Alkaloid
278.07	8a,12a-dihydro-8H-[1,3]dioxolo[4',5':4,5]benzo[1,2,3-de]benzo[g]quinolin-8-one	C_17_H_11_NO_3_		Alkaloid
354.13	1,2,9,10-tetramethoxy-3H-dibenzo[de,g]quinolin-7(5H)-one	C_20_H_19_NO_5_		Alkaloid
306.07	5-methoxy-8H-[1,3]dioxolo[4',5':4,5]benzo[1,2,3-de]benzo[g]quinolin-8-one	C_18_H_11_NO_4_		Alkaloid
338.10	9-hydroxy-1,2,3-trimethoxy-7H-dibenzo[de,g]quinolin-7-one	C_19_H_15_NO_5_		Alkaloid
579.17	Kaempferitrin	C_27_H_30_O_14_		Flavonoid
593.27	5-hydroxy-2-(4-methoxyphenyl)-7-(3,4,5-trihydroxy-6-methyloxan-2-yl)oxy-3-(3,4,5-trihydroxyoxan-2-yl)oxychromen-4-one	C_28_H_32_O_14_		Flavonoid
291.09	Catechin	C_15_H_14_O_6_		Flavonoid
287.06	Kaempferol	C_15_H_10_O_6_		Flavonoid
285.10	3,4-dimethoxy-4'-hydroxychalcone	C_17_H_16_O_4_		Flavonoid
329.10	5-hydroxy-3,7-dimethoxy-2-(4-methoxyphenyl)-4H-chromen-4-one	C_18_H_16_O_6_		Flavonoid
303.05	Quercetin	C_15_H_10_O_7_		Flavonoid
565.15	5-hydroxy-2-(4-hydroxyphenyl)-7-(3,4,5-trihydroxy-6-methyloxan-2-yl)oxy-3-(3,4,5-trihydroxyoxan-2-yl)oxychromen-4-one	C_26_H_28_O_14_		Flavonoid
533.16	Quercetin_7-glycosides_7-O-[2S-Methylbutanoyl-(3)-L-rhamnopyranoside]	C_26_H_28_O_12_		Flavonoid
551.14	7-Hydroxy-4'-methoxyisoflavone_7-O-[4-Hydroxybenzoyl-(2)-D-glucopyranoside]	C_29_H_26_O_11_		Flavonoid
357.10	3',4',5,7-Tetrahydroxy-8-prenylflavanone	C_20_H_20_O_6_		Flavonoid
165.05	*p*-coumaric acid	C_9_H_8_O_3_		Phenylpropanoid
149.04	Cinnamic acid	C_9_H_8_O_2_		Phenylpropanoid
149.08	Estragole	C_10_H_12_O		Phenylpropanoid
265.10	4,7,8-trimethoxy-3,5-dimethylchromen-2-one	C_14_H_16_O_5_		Coumarins
377.14	[1,3-dihydroxy-1-(7-methoxy-2-oxochromen-6-yl)-3-methylbutan-2-yl] (E)-2-methylbut-2-enoate	C_20_H_24_O_7_		Coumarins
189.05	5-Hydroxy-2-methyl-1,4-naphthoquinone	C_11_H_8_O_3_		Quinone

### Multivariate and univariate statistical analysis reveal cytotoxicity-driven metabolic discrimination

**Fig. 4:**
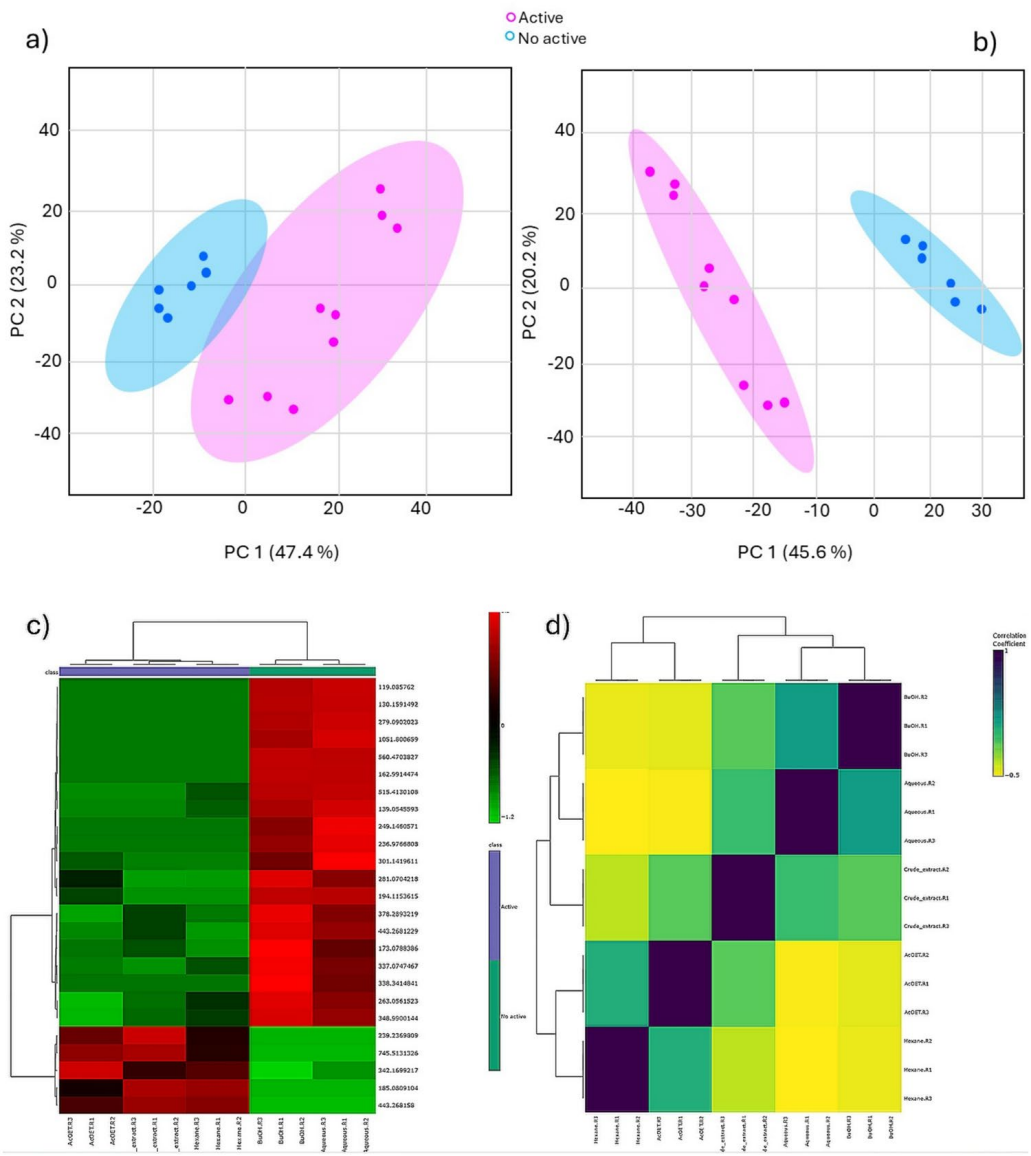
Multivariate statistical analysis of *Siparuna guianensis* fractions. **a** Principal Component Analysis (PCA) score plot showing the intrinsic metabolic organization and variance explained by PC1 and PC2. **b** Partial Least Squares–Discriminant Analysis (PLS-DA) demonstrating supervised separation between active and inactive groups. **c** Hierarchical clustering heatmap based on the top 25 significant features selected by ANOVA and *t*-test, highlighting differential metabolite abundance patterns. **d** Pearson correlation heatmap illustrating similarity among biological replicates and relationships between fractions according to metabolic composition

To investigate metabolomic variability among *S. guianensis* samples and identify chemical signatures associated with cytotoxic activity, complementary multivariate and univariate statistical approaches were applied, including unsupervised pattern recognition, supervised classification, hierarchical clustering, and correlation analyses (Fig. 4).

To further evaluate whether the observed separation was driven by cytotoxic activity rather than extraction solvent, additional exploratory analyses were performed. PCA and PLS-DA models were examined considering both biological classification (active vs inactive) and extraction solvent grouping. While solvent-based clustering was partially observed, supervised models consistently achieved stronger discrimination when class labels were defined based on cytotoxic activity, supporting the biological relevance of the observed metabolic signatures.

Unsupervised PCA analysis revealed a clear intrinsic organization of samples, with PC1 and PC2 explaining 47.4% and 23.2% of the total variance, respectively, accounting for 70.6% of cumulative variability (Fig. 4a). Notably, PCA clearly separated cytotoxic (active) and non-cytotoxic (inactive) fractions along PC1, indicating that biological activity correlates with dominant compositional gradients in the metabolome. Supervised analysis from PLS-DA further enhanced group discrimination, with PC1 and PC2 explaining 45.6% and 20.2% of the variance (65.8% cumulative), resulting in a pronounced separation between classes and supporting the presence of metabolomic variation associated with cytotoxic activity (Fig. 4b). Model validation metrics demonstrated high robustness and predictive reliability, with optimal performance achieved using four latent variables, where accuracy, R^2^, and Q^2^ simultaneously reached their highest values. Although the model showed high predictive performance (Q^2^ approaching unity), such values should be interpreted with caution due to the limited sample size and high dimensionality of metabolomics data, which may lead to overestimation of model robustness.

To identify statistically significant discriminatory metabolites, hierarchical clustering heatmaps based on the top 25 features selected by combined ANOVA and *t*-test filtering were constructed (Fig. 4c). The resulting clustering pattern revealed two major metabolic blocks corresponding to active and inactive samples, indicating consistent abundance patterns associated with biological activity following statistical feature selection. Cytotoxic fractions displayed coordinated enrichment of specific ion clusters, whereas polar fractions exhibited comparatively homogeneous metabolite profiles with reduced abundance of discriminatory features.

Correlation analysis among samples (Fig. 4d) reinforced these observations by demonstrating strong intra-fraction reproducibility and clear inter-fraction metabolic divergence. Hexane and ethyl acetate fractions exhibited higher similarity to the crude extract, forming a chemically coherent cluster consistent with their shared cytotoxic phenotype. In contrast, butanol and aqueous fractions showed stronger mutual correlation, reflecting enrichment in more polar metabolite pools and reduced association with bioactivity.

Collectively, these results demonstrate that both unsupervised and supervised statistical analyses converge toward a cytotoxicity-driven metabolomic organization, supporting the effectiveness of liquid–liquid partitioning in chemically segregating bioactive metabolite classes and enabling reliable biomarker discovery within complex plant metabolomes.

**Fig. 5:**
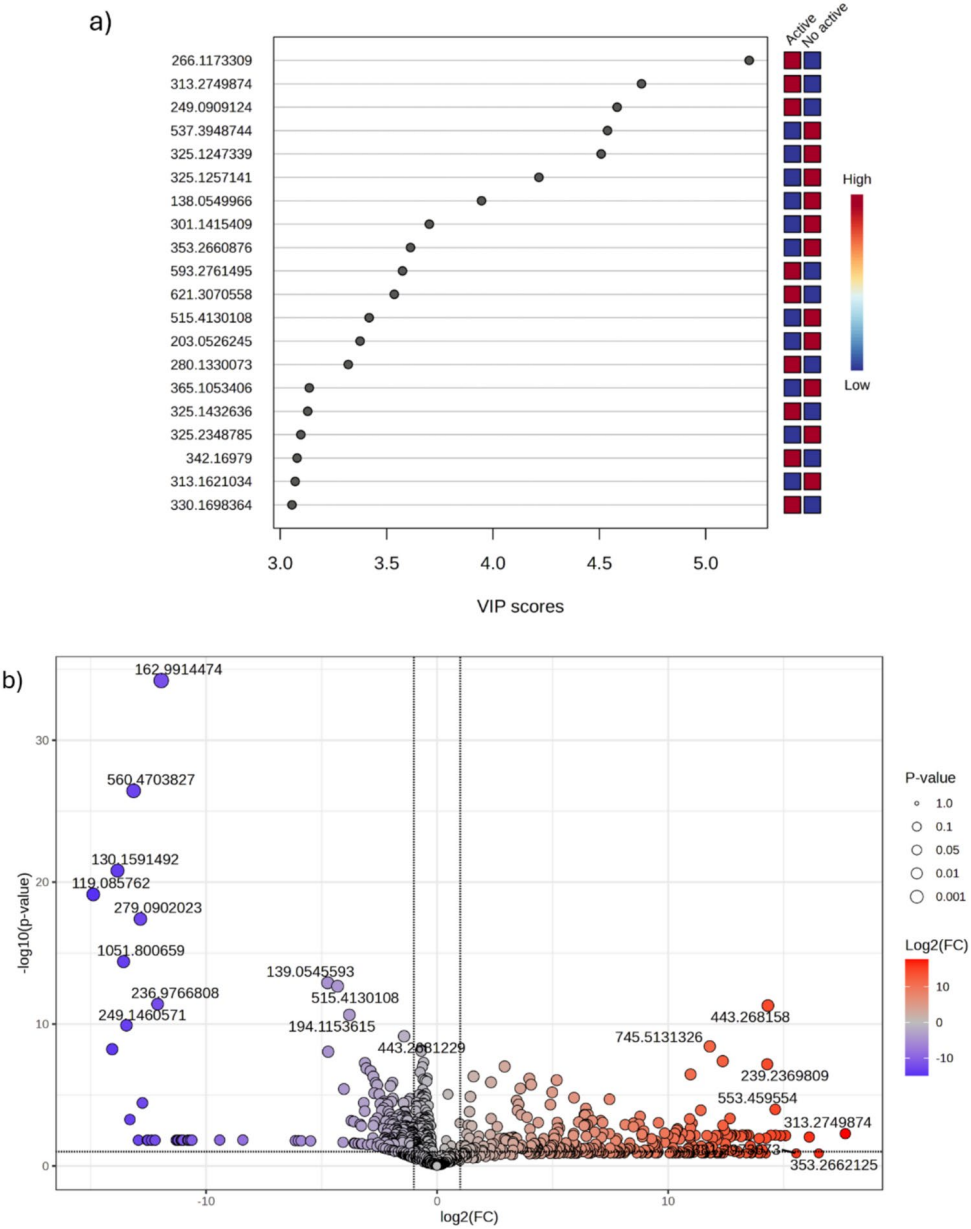
Integrated univariate and supervised multivariate statistical analyses highlighting the most discriminant metabolites between active and inactive fractions. **a** Variable Importance in Projection (VIP) scores obtained from the optimized PLS-DA model, showing the top 20 features contributing to group discrimination. **b** Volcano plot combining fold-change and statistical significance (t-test/ANOVA), emphasizing the most significantly altered ions between groups

The multivariate and univariate statistical analyses revealed a consistent metabolic discrimination between active and inactive fractions, which guided the identification of the most relevant chemical drivers associated with cytotoxic activity. Building upon the clear class separation observed in PCA and the highly predictive discrimination achieved by the optimized PLS-DA model, biomarker prioritization was performed using Variable Importance in Projection (VIP) scores combined with fold-change–based statistical filtering. Permutation tests confirmed that model performance was significantly higher than random classification (*p* < 0.01), although it does not exclude the influence of solvent-driven variation. As shown in Fig. 5a, the VIP analysis highlighted the metabolites with the greatest contribution to class separation, with all selected features presenting VIP values substantially above the conventional threshold (VIP > 1), indicating strong influence on the latent variables responsible for discriminating active and inactive samples. Notably, several ions exhibited exceptionally high VIP scores (> 3.0), reinforcing their relevance as potential chemical markers associated with bioactivity patterns. Complementarily, the Volcano plot (Fig. 5b) integrated statistical significance and magnitude of variation, combining ANOVA/t-test results with fold-change analysis to identify metabolites simultaneously exhibiting strong differential abundance and robust statistical confidence. Features positioned at the extremes of the distribution showed both elevated—log10(p-values) and pronounced log2(FC) deviations, confirming substantial metabolic remodeling between groups. Importantly, these discriminatory ions were predominantly associated with isoquinoline alkaloids annotated in this study, alongside polyphenolic metabolites, particularly glycosylated flavonoids and simple phenols, metabolite classes widely reported for their cytotoxic and redox-modulating properties.

The integration of univariate analysis (Volcano plot, combining Fold Change and *t*-tests) with supervised multivariate modeling (VIP scores from PLS-DA) led to the identification of a refined subset of consensus metabolites strongly associated with cytotoxic activity. The overlap between statistical significance and discriminative relevance yielded ten intersecting ions, representing the most robust bioactivity-associated features within the dataset.

**Fig. 6:**
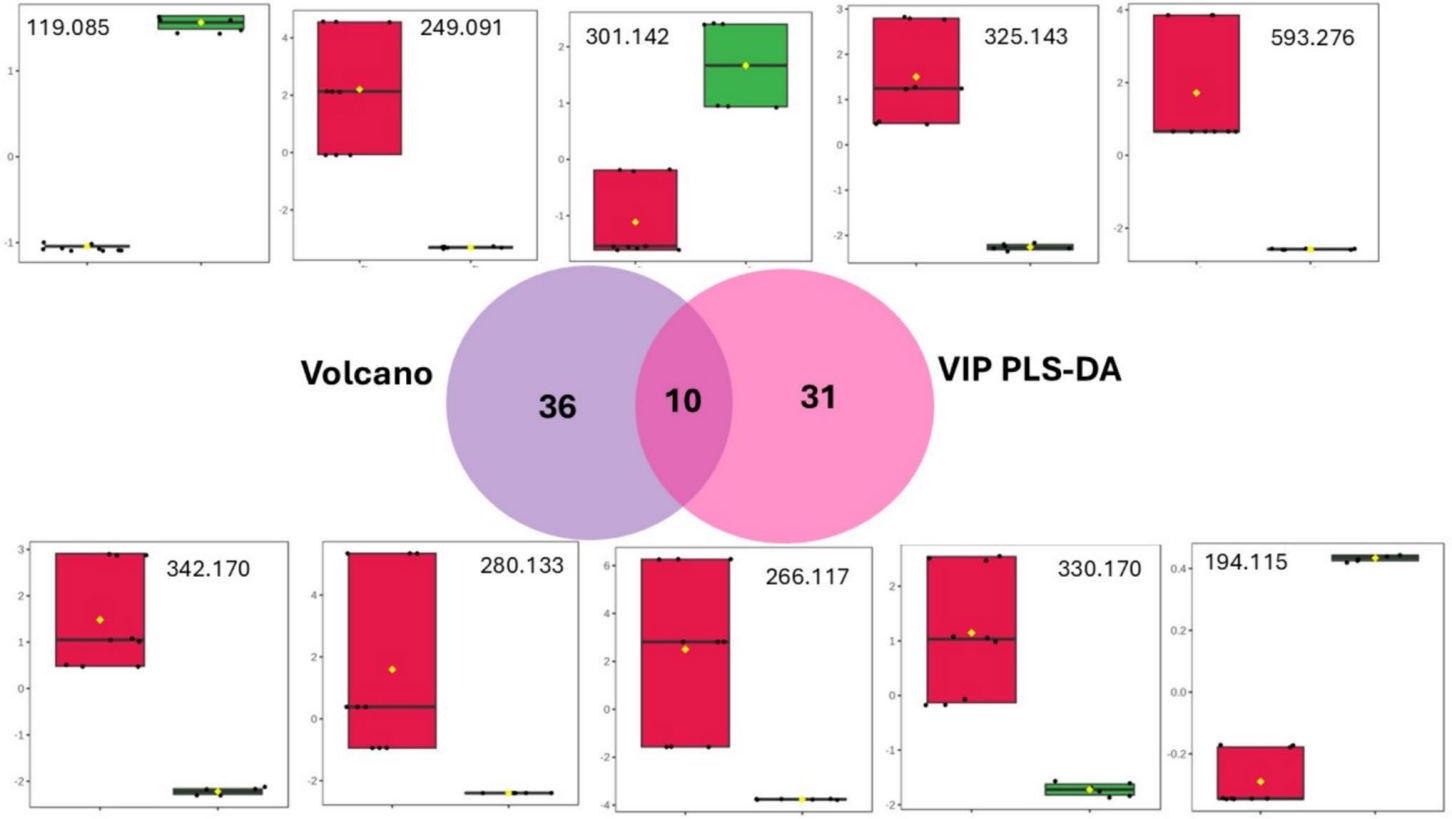
Venn diagram showing the overlap between significant features identified by the Volcano plot (Fold Change and *t*-test) and discriminant variables selected by VIP scores from the PLS-DA model. Box plots represent the normalized relative intensities of the intersecting ions (*m/z* 119.05, 249.091, 301.142, 325.143, 593.276, 342.170, 280.133, 266.117, 330.170, and 194.115) across the evaluated groups

The ions identified in the Volcano-VIP intersection were *m/z* 119.05, 249.091, 301.142, 325.143, 593.276, 342.170, 280.133, 266.117, 330.170, and 194.115 (Fig. 6). Structural annotation revealed that these features predominantly correspond to alkaloids and flavonoids, reinforcing the chemical basis of the observed cytotoxic phenotype. Notably, *m/z* 593.276 was annotated as a methoxylated kaempferitrin derivative, highlighting the contribution of flavonoid glycosides. Several isoquinoline and aporphine alkaloids were also identified, including *m/z* 342.170, assigned to Norglaucine; *m/z* 330.170, corresponding to 3-(3-hydroxy-4-methoxybenzyl)-7-methoxy-2-methyl-1,2,3,4-tetrahydroisoquinolin-6-ol; and *m/z* 194.115, identified as (S)-7-methoxy-1-methyl-1,2,3,4-tetrahydroisoquinolin-6-ol. Additionally, the aporphinic alkaloids *m/z* 280.133 (7-methyl-6,7,7a,8-tetrahydro-5H-[1,3]dioxolo[4',5':4,5]benzo[1,2,3-de]benzo[g]quinoline) and *m/z* 266.117 (6,7,7a,8-tetrahydro-5H-[1,3]dioxolo[4',5':4,5]benzo[1,2,3-de]benzo[g]quinoline) further support the prominence of isoquinoline-derived scaffolds within the active group.

Collectively, the convergence of statistical rigor and structural annotation indicates that isoquinoline and aporphine alkaloids, together with flavonoid derivatives, constitute the principal chemical drivers of cytotoxicity in *Siparuna guianensis*. These metabolites emerge as priority candidates for targeted isolation, quantitative validation, and mechanistic investigation, providing a plausible chemical basis for the bioactivity-guided metabolomic findings.

Notably, the ion at *m/z* 342.17, tentatively annotated as norglaucine, emerged as one of the most promising biomarkers in the intersection between VIP and volcano analyses. This finding is particularly compelling, as norglaucine has been previously reported to exhibit significant cytotoxic activity against multiple tumor cell lines, including B16-F10, HepG2, K562, and HL-60, with a strong IC₅₀ observed in at least one lineage (Menezes et al., 2016). The convergence of chemometric prioritization and independent biological evidence reinforces the relevance of this alkaloid as a potential contributor to the observed cytotoxicity and supports its consideration as a key candidate for further mechanistic and functional investigations.

Importantly, the annotation of norglaucine as a top-ranked discriminant metabolite provides additional biological support for the model, as this compound has been previously reported to exhibit cytotoxic activity. This convergence between statistical selection and independent literature evidence reinforces the biological relevance of the observed metabolomic patterns.

**Fig. 7:**
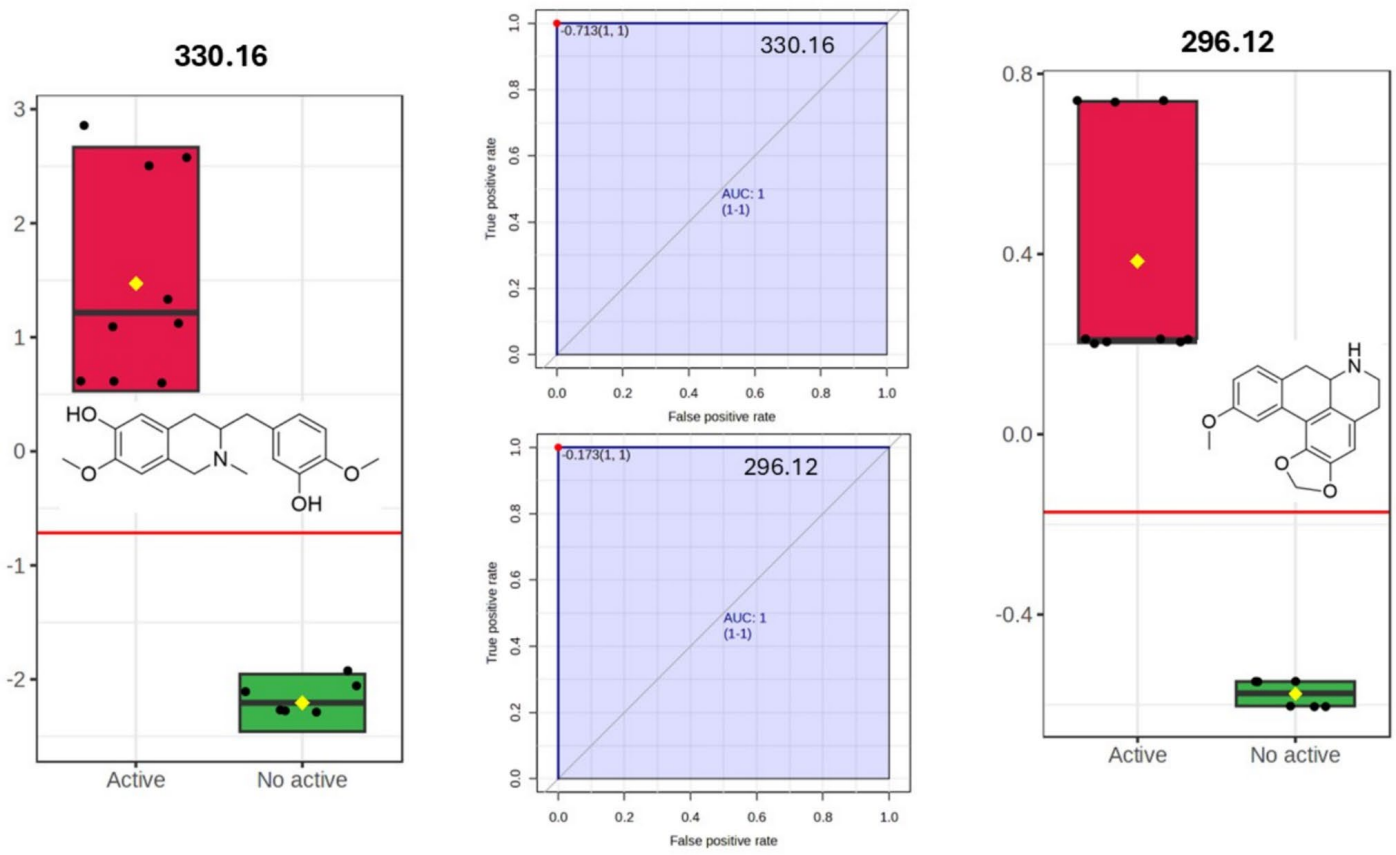
Biomarker analysis integrating ROC curve performance (AUROC), T-statistics, and Log₂ fold change (FC) for feature prioritization. Box plots display the normalized relative intensities of the top-ranked ions (*m/z* 330.170 and 296.12) across active and inactive groups. Corresponding ROC curves illustrate their classification performance. Structural representations of the annotated alkaloids are shown within the panels

Consistent with the previously identified consensus ions derived from the integration of Volcano and VIP analyses, biomarker prioritization based on AUROC, T-statistics, and Log₂ fold change further refined the set of metabolites most strongly associated with cytotoxic activity (Fig. 7). Among the ranked features, *m/z* 330.170 and *m/z* 296.12 emerged as the most discriminant variables. The ion at *m/z* 330.170, annotated as the benzyl-tetrahydroisoquinoline alkaloid 3-(3-hydroxy-4-methoxybenzyl)-7-methoxy-2-methyl-1,2,3,4-tetrahydroisoquinolin-6-ol, and *m/z* 296.12, identified as the aporphinic alkaloid 11-methoxy-6,7,7a,8-tetrahydro-5H-[1,3]dioxolo[4',5':4,5]benzo[1,2,3-de]benzo[g]quinoline, displayed the highest classification performance according to ROC analysis and effect size metrics. Their elevated AUROC values suggest strong discriminative ability, although these results should be interpreted cautiously due to the limited sample size. Notably, both compounds belong to the isoquinoline-derived alkaloid family, reinforcing the central contribution of benzylisoquinoline and aporphine scaffolds to the cytotoxic profile of *Siparuna guianensis* and highlighting these structures as prime candidates for targeted isolation and functional validation.

**Fig. 8:**
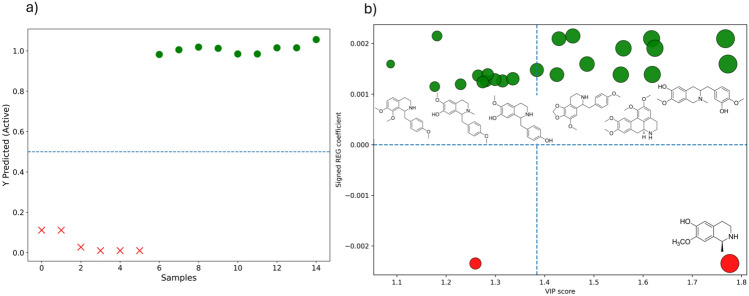
**a** PLS-DA prediction model built using OPSDA-selected variables showing robust discrimination between cytotoxic (active) and inactive samples across the decision threshold (dashed line). **b** Alkaloid activity landscape displaying VIP scores versus signed regression (REG) coefficients. VIP indicates statistical importance, whereas signed REG coefficients describe biological association with cytotoxic (green) or inactive (red) samples. Bubble size reflects relative abundance, and arrows highlight the top ten biomarkers ranked by VIP ×|REG|. Dashed lines indicate the active–inactive boundary (REG = 0) and the median VIP threshold separating highly influential metabolites. Chemical structures of prioritized alkaloids are shown within their respective quadrants

To refine biomarker discovery, a variable-selected PLS-DA model was constructed using Ordered Predictors Selection for Discriminant Analysis (OPSDA). Model robustness was ensured through stratified five-fold cross-validation. The model demonstrated outstanding discriminative capability between cytotoxic (active) and non-cytotoxic (inactive) samples, achieving near-complete class separation. Predicted response values (ŷ) exhibited a clear bimodal distribution, with active samples consistently clustering above the classification threshold and inactive samples remaining markedly below it, indicating high sensitivity, specificity, and reproducibility across validation folds. Figure 8a illustrates the classification performance obtained using OPSDA-selected variables, confirming the preservation of predictive accuracy with a reduced metabolite subset. Figure 8b presents the combined Variable Importance in Projection (VIP) and regression (REG) scores for isoquinoline alkaloids selected by OPSDA. While VIP reflects the overall statistical importance of each variable in explaining model variance, REG coefficients quantify the direct contribution and biological directionality toward class prediction. Positive REG coefficients (green) indicate alkaloids enriched in cytotoxic samples and potentially associated with cytotoxic activity, whereas negative coefficients (red) characterize metabolites enriched in inactive extracts.

Biomarkers were prioritized using the combined VIP ×|REG| metric, with arrow annotations highlighting the top ten discriminant alkaloids. In this representation, VIP conveys statistical relevance, signed REG coefficients indicate biological association, and bubble size reflects relative metabolite abundance. The mechanistic quadrant map further revealed a subset of alkaloids simultaneously exhibiting high VIP scores and positive regression coefficients, suggesting a central contribution to the cytotoxic phenotype. Conversely, metabolites displaying high VIP values, but negative REG coefficients may represent antagonistic or counteracting chemical signatures linked to inactive samples. Chemical structures of the top-ranked alkaloids were assigned within their respective quadrants, enabling direct structure–activity interpretation. The horizontal axis delineates the active versus inactive decision boundary, whereas the vertical axis corresponds to the median VIP threshold separating highly influential from low-importance variables.

## Conclusion

This study demonstrates that the cytotoxic activity of *Siparuna guianensis* suggests that cytotoxic activity is associated with metabolomic composition. Bioactivity-guided fractionation combined with LC–HRMS untargeted metabolomics showed that low- and intermediate-polarity fractions concentrate the main cytotoxic chemical space, while polar fractions retain chemically distinct but less active metabolite pools. Spectral comparison and feature intersection analyses confirmed the efficiency of liquid–liquid partitioning in generating chemically orthogonal fractions suitable for metabolomic discrimination.

MSI level 2 annotations supported by GNPS and SIRIUS workflows revealed a metabolome enriched in isoquinoline-derived alkaloids and flavonoids, which were proportionally more abundant in cytotoxic samples despite the overall dominance of terpenoids. Multivariate and univariate statistical analyses consistently demonstrated activity-driven metabolic segregation, with PCA and PLS-DA models providing robust discrimination and predictive reliability. Additionally, integration of VIP scores, Volcano analysis, and ROC-based prioritization identified isoquinoline and aporphine alkaloids, together with glycosylated flavonoids, as the principal chemical drivers of cytotoxicity. The identification of norglaucine as a top discriminant feature reinforces its biological relevance, consistent with its previously reported cytotoxic activity across multiple tumor cell lines and highlights its potential role as a key driver of the observed anticancer effects. These findings establish a direct link between chemical composition and biological activity in *S. guianensis*, highlighting isoquinoline-derived scaffolds as priority targets for future isolation and mechanistic investigation, while demonstrating the effectiveness of metabolomics-guided strategies for accelerating natural product discovery.

Despite the clear separation observed, it is important to acknowledge that solvent polarity contributes significantly to metabolite distribution. Therefore, part of the observed clustering may reflect extraction-driven chemical differences. The integration of biological classification (active vs inactive) and feature selection strategies partially mitigates this effect; however, further validation using larger datasets and independent samples is required.

## Data Availability

No datasets were generated or analysed during the current study.
